# Risk factors on admission associated with hospital length of stay in patients with COVID-19: a retrospective cohort study

**DOI:** 10.1038/s41598-021-86853-4

**Published:** 2021-03-31

**Authors:** Anping Guo, Jin Lu, Haizhu Tan, Zejian Kuang, Ying Luo, Tian Yang, Junlan Xu, Jishuang Yu, Canhong Wen, Aizong Shen

**Affiliations:** 1 Department of Pharmacy, The First Affiliated Hospital of USTC, Division of Life Sciences and Medicine, University of Science and Technology of China, Hefei, Anhui 230001 People’s Republic of China; 2 Shantou University Medical College, Shantou, Guangdong People’s Republic of China; 3 The Second Affiliated Hospital of Shantou University Medical College, Shantou, Guangdong People’s Republic of China; 4 Infectious Diseases Department of the First Affiliated Hospital, University of Science and Technology of China, Hefei, Anhui People’s Republic of China; 5 Department of Statistics and Finance, School of Management, University of Science and Technology of China, Hefei, 230026 Anhui People’s Republic of China

**Keywords:** Risk factors, Viral infection

## Abstract

Treating patients with COVID-19 is expensive, thus it is essential to identify factors on admission associated with hospital length of stay (LOS) and provide a risk assessment for clinical treatment. To address this, we conduct a retrospective study, which involved patients with laboratory-confirmed COVID-19 infection in Hefei, China and being discharged between January 20 2020 and March 16 2020. Demographic information, clinical treatment, and laboratory data for the participants were extracted from medical records. A prolonged LOS was defined as equal to or greater than the median length of hospitable stay. The median LOS for the 75 patients was 17 days (IQR 13–22). We used univariable and multivariable logistic regressions to explore the risk factors associated with a prolonged hospital LOS. Adjusted odds ratios (aORs) and 95% confidence intervals (CIs) were estimated. The median age of the 75 patients was 47 years. Approximately 75% of the patients had mild or general disease. The univariate logistic regression model showed that female sex and having a fever on admission were significantly associated with longer duration of hospitalization. The multivariate logistic regression model enhances these associations. Odds of a prolonged LOS were associated with male sex (aOR 0.19, 95% CI 0.05–0.63, p = 0.01), having fever on admission (aOR 8.27, 95% CI 1.47–72.16, p = 0.028) and pre-existing chronic kidney or liver disease (aOR 13.73 95% CI 1.95–145.4, p = 0.015) as well as each 1-unit increase in creatinine level (aOR 0.94, 95% CI 0.9–0.98, p = 0.007). We also found that a prolonged LOS was associated with increased creatinine levels in patients with chronic kidney or liver disease (p < 0.001). In conclusion, female sex, fever, chronic kidney or liver disease before admission and increasing creatinine levels were associated with prolonged LOS in patients with COVID-19.

## Introduction

Starting in December 2019, a novel coronavirus, designated SARS-CoV-2, caused an international outbreak of respiratory disease termed COVID-19^1^. Up to April 7, 2020, a total of 1,279,722 laboratory-confirmed cases were reported worldwide, including at least 72,614 deaths^2^. Because of an increased potential to cause severe disease and a high transmissibility, COVID-19 was declared a “public health emergency of international concern” by the World Health Organization on January 30, 2020^3^. COVID-19 also exacerbates economic and social costs beyond mortality and morbidity, which can persist and worsen^4^.

For healthcare system implications, many doctors, nurses and hospital beds are needed to treat patients with confirmed disease, leading to overburdened hospitals and overtaxed medical resources around the world. Furthermore, the median length of hospital stay (LOS) among COVID-19 survivors was found to be 10 to 13 days^5–7^. In China, the average total cost per patient who requires inpatient stay is 17,000 yuan^8, 9^ or $73,300 USD^10^. Thus, studying the admission risk factors for LOS and identifying patients with prolonged LOS early are important. In this way, clinicians could attend to these patients more closely to help reduce their LOS and consequently decrease healthcare costs.

A number of related studies have been reported for other contagious diseases, such as Middle East respiratory syndrome (MERS) and H1N1 flu. MERS causes a substantial health burden in Saudi Arabia due to its high morbidity, high hospitalization rates, long hospital stays, and shortages of staff^11^. In a 1-year national retrospective chart review of all patients with laboratory-confirmed H1N1 influenza in the United States, age and days to antiviral initiation were independently associated with prolonged disease^12^. As far as the authors are aware, there are several related works on COVID-19. For example, Liu et al. investigated the risk factors associated with disease severity and LOS in hospitals for COVID-19 patients with moderate or severe disease status and found that glucocorticoid use resulted in a prolonged LOS^13^. Another study focused on asymptomatic patients and those with mild symptoms^14^, where the identified risk factors included fever before admission to the hospital and bilateral pneumonia. In addition to China, a retrospective study in Vietnam showed that age, residence, and sources of contamination were significantly associated with a longer duration of hospitalization in patients with COVID-19 during the second wave of COVID-19^15^. The first two studies considered only some disease severity statuses, while nearly 40% of patients were still hospitalized in the last study. Very recently, there was a systematic review and meta-analysis concerning LOS of patients with COVID-19 referencing 52 studies worldwide^16^. The review provided summary LOS distributions by combining information from different summary statistics and accounting for differences in sample sizes. Substantial differences were found between China and other locations, yet little evidence was found to impact LOS. The main reason might be the heterogeneity of LOS in the patients; this finding may be attributed to patient and hospital characteristics^17^.

To explore the hospital admission risk factors associated with LOS, we conducted a retrospective study of patients hospitalized with COVID-19 from a single hospital in Hefei, China. We included all the patients from this hospital who were discharged after satisfying the discharge criteria, and all kinds of disease severity statuses were considered.

## Methods

### Study design and patients

A retrospective study was conducted for patients with a COVID-19 diagnosis according to the “Diagnosis and Treatment Scheme for Novel Coronavirus Pneumonia (Trial), 6th Edition” enacted by the National Health Commission of the People’s Republic of China on February 19, 2020^18^ and who were discharged from January 20, 2020 to March 16, 2020 from the Infectious Diseases Department of the First Affiliated Hospital of the University of Science and Technology of China. All patients received antiviral treatment with lopinavir and ritonavir (400 mg twice daily and 100 mg twice daily, respectively). The discharge criteria were adopted from the “Diagnosis and Treatment Scheme for Novel Coronavirus Pneumonia (Trial), 6th Edition” in China: (1) Temperature returned to normal for more than 3 days; (2) There was significant improvement in respiratory symptoms; (3) Pulmonary imaging showed significant improvement in acute exudative lesions; and (4) The PCR sample remained negative for at least 24 h. We only included patients with normal discharge circumstances and excluded those with death outcomes.

The study was approved by the Research Ethics Commission of the First Affiliated Hospital of the University of Science and Technology of China (2020-P-018), and the requirement for informed consent was waived by the ethics commission. All methods were performed in accordance with the relevant guidelines and regulations.

### Data collection

Epidemiological, demographic, clinical, and laboratory data for each patient were extracted from the electronic medical records by using a standardized data collection form. The severity of COVID-19 was defined according to the Chinese management guidelines for COVID-19 (version 6.0). Symptoms and other epidemiological features on hospital admission were recorded. Blood testing was performed within 72 h after admission and included complete blood count, kidney function, liver function and levels of C-reactive protein, creatinine kinase, lactate dehydrogenase, D-dimer, CD4/CD8, electrolytes, myocardial enzymes, interleukin-6, triglycerides, glucose, procalcitonin and complete bilirubin. All patients underwent chest radiography or CT scans.

The median LOS for the 75 patients was 17 days (IQR 13–22) (range 4 to 34 days). Therefore, we defined a hospital stay less than the median LOS as a normal LOS and a stay longer than the median as prolonged LOS.

### Statistical analysis

Continuous and categorical variables are presented with medians (interquartile ranges) and numbers (%), respectively. We used the Mann–Whitney U test or Fisher’s exact test to compare differences in LOS. Missing data for each laboratory test imputed with the median value. A univariable logistic analysis was used to obtain unadjusted odds ratios (ORs) with 95% confidence intervals (CIs). A multivariable logistic regression model was used to evaluate the adjusted odds of association with LOS. To determine the relevant predictor variables and avoid overfitting in the model, we chose the best model with the fewest variables while maintaining the prediction accuracy by using the best subset selection method with the Akaike information criterion (AIC) Akaike^19^. The final multivariable logistic regression model was established by the AIC best subset selection approach^20^. We report adjusted ORs (aORs) with 95% CIs for each predictor variable.

For the identified laboratory variables, we performed repeated-measures ANOVA to study the day-to-day change from admission to 15 days of hospitalization. Variables with statistically significant associations with prolonged LOS in multivariable analyses were also used. These variables included sex, fever, and chronic kidney or liver disease on admission. We also included an interaction term between chronic kidney or liver disease and creatinine level in the repeated-measures ANOVA.

All statistical analyses were performed with R software with version v3.6.1, and p < 0.05 was considered statistically significant.

### Role of the funding source

The funder of the study had no role in the study design, data collection, data analysis, data interpretation, or writing of the report. The corresponding authors (CW and AS) had full access to all the data in the study and had final responsibility for the decision to submit for publication.

## Results

We initially included 84 patients; however, seven patients were excluded because of participation in another clinical trial or because of death, and two patients were excluded because of incomplete medical record information. Therefore, 75 patient records were used in the study. The characteristics for the patients are listed in Table 1. The median age of patients was 47 (IQR 31–54) years (range 5–91), and 57% were male. Approximately three-quarters of the patients had mild or general disease (Table 1). About half of the patients had an exposure history to Wuhan. In total, 12 (16%) patients were transferred from other hospitals, and the remaining patients were first diagnosed at the First Affiliated Hospital. Only three patients were current smokers. Comorbidity was present in more than one-third of the patients, with hypertension being the most common comorbidity, followed by diabetes (Table 1). The most common symptoms on admission were fever and cough, followed by chest congestion and fatigue. The most common features determined by CT were bilateral pulmonary infiltration (89%), ground-glass opacities (43%) and consolidation (17%). The hospital LOS was longer for patients with fever than for those without fever on admission (p = 0.042), i.e., the median LOS of patients with fever was 21 days and that of patients without fever was 12.5 days.

**Table 1:** Epidemiological, demographic, clinical and laboratory characteristics of COVID-19 patients by hospital length of stay (LOS).

	Total (n = 76)	Hospital LOS < 17 days (n = 36)	Hospital LOS ≥ 17 days (n = 39)	p value
**Demographic and clinical characteristics**				
Disease severity status				0.797
Mild or general	54 (72%)	25 (69%)	29 (74%)	
Severe or critical	21 (28%)	11 (31%)	10 (26%)	
Sex				0.061
Female	32 (43%)	11 (31%)	21 (54%)	
Male	43 (57%)	25 (69%)	18 (46%)	
Age, years, median (IQR) (range)	47 (31–54) (5–91)	43.5 (29.8–52.3)	47 (33.5–56)	0.644
Exposure history to Wuhan	41 (55%)	18 (50%)	23 (59%)	0.491
Source of patients				0.212
First diagnosis	63 (84%)	28 (78%)	35 (90%)	
Transfer from other hospitals	12 (16%)	8 (22%)	4 (10%)	
Smoker	3 (4%)	2 (6%)	1 (3%)	0.605
Fever	65 (87%)	28 (78%)	37 (95%)	0.042
Cough	47 (63%)	22 (61%)	25 (64%)	0.815
Myalgia	4 (5%)	2 (6%)	2 (5%)	1
Throat pain	4 (5%)	2 (6%)	2 (5%)	1
Fatigue	10 (13%)	7 (19%)	3 (8%)	0.181
Chest congestion	11 (15%)	4 (11%)	7 (18%)	0.520
Comorbidity	32 (42%)	14 (34%)	18 (51%)	0.160
Hypertension	13 (17%)	7 (19%)	6 (15%)	0.763
Diabetes	9 (12%)	5 (14%)	4 (10%)	0.730
Chronic kidney or liver disease	10 (13%)	4 (11%)	6 (15%)	0.738
**Laboratory characteristics in the first 2 days after in hospital**				
CRP, mg/L	10.3 (2.5–27.7)	14.3 (2.1–28.1)	8.2 (2.9–19.4)	0.695
White blood cell count, *10^9^/L	5.5 (4.7–7.7)	5.2 (4.7–7.7)	6.1 (4.8–7.5)	0.645
Lymphocyte count, *10^9^/L	1.3 (0.9–1.8)	1.3 (0.9–1.8)	1.4 (0.9–1.8)	0.869
Neutrophil count, *10^9^/L	3.6 (2.7–6.0)	3.4 (2.6–5.4)	3.8 (2.8–6.2)	0.494
D-dimer, µg/ml	0.2 (0.2–0.3)	0.2 (0.2–0.4)	0.2 (0.2–0.3)	0.341
CD4/CD8	1.4 (1.2–1.8)	1.4 (1.1–1.5)	1.4 (1.3–1.8)	0.272
AST, IU/L	24.2 (19.8–30.0)	23.5 (18.9–29.0)	24.2 (20.5–31.0)	0.567
ALT, IU/L	27.7 (16.9–36.8)	28.4 (16.0–32.5)	27.7 (19.3–39.3)	0.524
Creatinine, µmol/L	65.0 (58.2–72.8)	68.8 (60.9–78.0)	64.0 (53.8–70.2)	0.052
Procalcitonin, ng/ml	0.1 (0.1–0.2)	0.1 (0.1–0.2)	0.1 (0.1–0.2)	0.199
IL-6, pg/ml	5.7 (5.1–6.3)	5.7 (5.5–6.1)	5.7 (5.0–6.3)	0.431
Glucose, mmol/L	6.1 (5.3–7.5)	5.7 (5.4–7.7)	6.3 (5.2–7.4)	0.616
Lactate dehydrogenase, U/L	222.0 (178.6–271.7)	224.3 (184.2–273.5)	208.5 (177.3–263.2)	0.436
Troponin, µg/L	0.1 (0.1–0.3)	0.1 (0.1–0.3)	0.1 (0.1–0.3)	0.865
Total bilirubin, µmol/L	16.1 (12.1–22.4)	16.1 (12.8–21.2)	17.4 (12.0–22.9)	0.932
Indirect bilirubin, µmol/L	10.6 (7.7–13.6)	10.5 (7.3–12.3)	10.9 (7.8–13.6)	0.762
Direct bilirubin, µmol/L	6.3 (4.5–9.1)	6.3 (4.8–9.4)	6.0 (4.4–9.0)	0.504
**CT scan features**				
Ground-glass opacities	32 (43%)	12 (33%)	20 (51%)	0.161
Bilateral pulmonary infiltration	67 (89%)	31 (86%)	36 (92%)	0.469
Consolidation	13 (17%)	7 (19%)	6 (15%)	0.763

Data are median (IQR), or number (%). p values were calculated by Mann–Whitney U test or Fisher exact test, as appropriate.

*CRP* C-reactive protein, *IL-6* interleukin 6, *AST* aspartate aminotransferase, *ALT* alanine aminotransferase, *IQR*, inerquartile range.

In the univariable analysis, male sex and having a fever on admission were significantly associated with prolonged LOS: OR 0.38, 95% CI 0.14–0.96, p = 0.04; and OR 5.29, 95% CI 1.21–36.88, p = 0.04. A 1-unit (µmol/L) increase in creatinine level was related to a prolonged LOS (OR 0.97, 95% CI 0.94–1, p = 0.07), but this effect was weak.

The final logistic regression model, based on variable selection according to the AIC best-subset selection method, contained the predictor variables of sex, transfer from another hospital, fever or chest symptoms, bilateral patchy shadowing on CT, pre-existing chronic kidney or liver disease, creatinine level, and indirect bilirubin level. After controlling for all variables, the association between LOS and sex was enhanced: aOR 0.19, 95% CI 0.05–0.63, p = 0.01 (Table 2). A similar phenomenon was found for fever, pre-existing chronic kidney or liver disease, and creatinine levels. Odds of a prolonged LOS were associated with fever on admission (aOR 8.27, 95% CI 1.47–72.16, p = 0.028) and pre-existing chronic kidney or liver disease (aOR 13.73, 95% CI 1.95–145.4, p = 0.015) as well as each 1-unit increase in creatinine level (aOR 0.94, 95% CI 0.9–0.98, p = 0.007).

**Table 2:** Risk factors associated with prolonged hospital LOS.

	OR (95%)	p value	Adjusted OR (95%)	p value
**Demographic and clinical characteristics**				
Disease severity status	0.78 (0.28–2.16)	0.64	–	
Male sex (vs female)	0.38 (0.14–0.96)	0.04	0.19 (0.05–0.63)	0.010
Age, years	1.01 (0.98–1.03)	0.64	–	
Exposure history to Wuhan	1.44 (0.58–3.62)	0.44	–	
Source of patients	2.50 (0.71–10.17)	0.17	5.57 (0.88–47.14)	0.084
Smoker (vs non-smoker)	0.45 (0.02–4.87)	0.52	–	
Fever	5.29 (1.21–36.88)	0.04	8.27 (1.47–72.16)	0.028
Cough	1.14 (0.44–2.92)	0.79	–	
Myalgia	0.92 (0.11–8.00)	0.93	–	
Throat pain	0.92 (0.11–8.00)	0.93	–	
Fatigue	0.35 (0.07–1.36)	0.15	–	
Chest congestion	1.75 (0.48–7.23)	0.41	4.01 (0.69–32.96)	0.151
**Comorbidity**				
Hypertension	0.75 (0.22–2.52)	0.64	–	
Diabetes	0.71 (0.16–2.91)	0.63	–	
Chronic kidney or liver disease	1.45 (0.38–6.14)	0.59	13.73 (1.95–14,540.)	0.015
**Laboratory characteristics in the first 2 days in hospital**				
CRP, mg/L	1.00 (0.98–1.02)	0.88	–	
White blood cell count, *10^9^/L	0.99 (0.82–1.19)	0.90	–	
Lymphocyte count, *10^9^/L	0.98 (0.52–1.83)	0.95	–	
Neutrophil count, *10^9^/L	1.01 (0.83–1.22)	0.94	–	
D-dimer, µg/ml	0.69 (0.22–1.66)	0.43	–	
CD4/CD8	0.96 (0.55–1.67)	0.89	–	
AST, IU/L	1.00 (0.97–1.03)	0.99	–	
ALT, IU/L	1.00 (0.99–1.02)	0.91	–	
Creatinine (per unit increase), µmol/L	0.97 (0.94–1.00)	0.07	0.94 (0.90–0.98)	0.007
Procalcitonin, ng/ml	0.72 (0.02–20.28)	0.83	–	
IL-6, pg/ml	0.99 (0.93–1.00)	0.55	–	
Glucose, mmol/L	1.00 (0.87–1.15)	0.97	–	
lactate dehydrogenase, U/L	1.00 (0.99–1.00)	0.73	–	
Troponin, µg/L	1.42 (0.39–6.72)	0.60	–	
Total bilirubin, µmol/L	1.00 (0.94–1.06)	0.93	–	
Indirect bilirubin, µmol/L	1.01 (0.91–1.12)	0.84	1.10 (0.97–1.25)	0.162
Direct bilirubin, µmol/L	1.00 (0.98–1.02)	0.88	–-	
**CT scan features**				
Ground-glass opacity	0.97 (0.83–1.11)	0.63	–	
Bilateral patchy shadowing	2.11 (0.84–5.47)	0.12	3.46 (1.02–13.31)	0.055
Patchy consolidation	1.94 (0.44–10.05)	0.39	–	

*OR* odds ratio, *95% CI* 95% confidence intervals.

**Figure 1:**
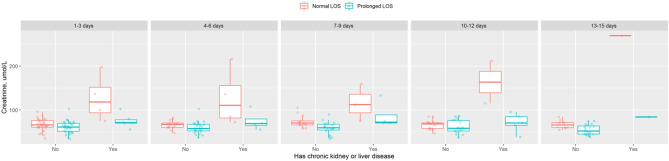
Creatinine level during hospitalization period until 15 days.

Creatinine level was the only identified laboratory feature and the most significant risk factor associated with prolonged LOS, and we provide further insight into this variable by tracking its values from admission to 15 days after admission (Fig. 1). For patients without chronic kidney or liver disease, most creatinine values were in the normal range (41–81 µmol/L). A prolonged LOS was associated with increased creatinine levels, especially for patients with kidney or liver disease present at admission. This finding was confirmed by the results from the repeated-measures ANOVA (Table 3) (p < 0.001). Furthermore, we found an interaction effect between prolonged LOS and chronic kidney or liver disease (p < 0.001).

**Table 3:** Repeated-measures ANOVA of creatinine level.

	Df	Sum Sq	Mean Sq	F value	Pr(> F)
Male sex (vs female)	1	362,916.	362,916.	9.13	00,028.
Fever	1	96,728.	96,728.	2.43	01,202.
Chronic kidney or liver disease	1	4,297,213.	4,297,213.	10,808.	< 0.0001
Prolonged LOS (vs normal LOS)	1	1,757,889.	1,757,889.	44.21	< 0.0001
Chronic kidney or liver disease: prolonged LOS (vs normal LOS)	1	1,975,593.	1,975,593.	49.69	< 0.0001
Residuals	223	8,866,697.	39,761.		

## Discussion

Recent studies have investigated the risk factors for prolonged LOS in patients hospitalized with COVID-19. Our study included all types of disease severity patients in China. Moreover, the patients in our study had a complete follow-up period; that is, all of the patients were discharged from the hospital after satisfying the discharge criteria. We found that the odds of having a prolonged LOS were associated with female sex, fever and chronic kidney or liver disease, and increased creatinine levels. In addition, bilateral pulmonary infiltration was more frequent with prolonged LOS than with normal LOS. Further longitudinal analysis of increased creatinine levels validated its association with prolonged LOS.

In a meta-analysis of 50,466 patients with COVID-19, fever was the most common symptom (89.1%)^21^. In agreement with a previous study, 87% of our 75 patients had fever on admission, and these patients frequently had a prolonged LOS. In a recent study of 45 fatal cases of COVID-19, the main symptom was fever^22^. Chen et al. reported that high fever was associated with the development of acute respiratory distress syndrome in COVID-19 patients^23^. In a study with asymptomatic patients and those with mild symptoms, Wu et al. also found that having a fever before admission was associated with a prolonged LOS^14^. These studies indicate that fever is associated with increased illness severity and adverse outcomes, which could explain why patients with fever have a prolonged LOS. Similar to patients with H1N1, another highly infectious disease, COVID-19 patients presented with fever, cough, and dyspnea, but hemoptysis was less common^24^. One study of patients with H1N1 found bacterial coinfection, which is commonly associated with fever due to the pathogens that colonize the nasopharynx, such as Staphylococcus aureus, Streptococcus pneumonia, and Streptococcus pyogenes^25^.

COVID-19 patients in China were more frequently male (60%) than female^26^, which was also found in the current study. However, this study found that females were more likely than males to have a prolonged LOS. A recent retrospective study also found a significantly higher frequency of women than men among older patients, who had higher rates of severe/critical disease and shortness of breath than other ages^27^. Similarly, males are more susceptible to SARS-CoV and MERS-CoV infection than females^28^. With SARS, the entry of the coronavirus into the cell depends on ACE2, and a significantly higher ACE2 content was detected in older female rats than in male rats^29^. Genetic and molecular disparities between men and women have a role in the differing incidence, pathophysiology, clinical signs, and treatment outcomes of several cancers. Sex differences in cancer incidence are attributed to regulation at the genetic/molecular level as well as the activity of sex hormones, which in turn modulate gene expression in various cancers^27^. Our results suggest that females are more frequently infected by SARS-CoV-2 and require more time to recover from the disease than males. Further research is needed to investigate the pathogenesis of COVID-19 caused by sex differences.

Our results found that longer LOS was associated with mild diseases. One explanation could be that asymptomatic and symptomatic patients have similar viral loads as reported previously (SARS-CoV-2 Viral Load in Upper Respiratory Specimens of Infected Patients). According to the “Diagnosis and Treatment Scheme for Novel Coronavirus Pneumonia (Trial), 6th Edition” in China, patients with mild disease status were treated with a few symptomatic drugs and were mainly isolated from others. However, most of these patients still had a high viral load during hospitalization. We think this is the main reason why patients with mild disease might have a long LOS.

Our results showed increased odds of a prolonged LOS with chronic kidney or liver disease. Previously, chronic kidney disease was found to be the most common comorbidity in critically ill patients with COVID-19. Furthermore, patients with chronic kidney disease are more at risk for pneumonia than the general population^30^. After adjusting for confounders such as chronic kidney or liver disease, the odds of an extended LOS were associated with each 1-unit increase in creatinine level, which was reduced, although not significantly, on admission for patients with a prolonged LOS. This finding has several explanations. First, creatinine generation is decreased in the condition of low muscle mass, which is related to age and sex^31^. More specifically, females often have less muscle mass than males, and aging is also associated with decreased muscle mass^31^. In our study, more females than males had a prolonged LOS, and the reverse finding was observed in patients with a normal LOS. The average age of patients with a prolonged LOS was higher than that of patients with a normal LOS. In a large retrospective cohort study of 11,291 patients admitted to intensive care units, low baseline serum creatinine levels were associated with an increased risk of mortality in critically ill patients^32^. Thus, low serum creatinine levels may be a risk factor for an extended LOS with COVID-19.

Our study has some limitations. First, because of the retrospective study design, not all laboratory tests were performed for all patients. We imputed missing data with the corresponding median value, which might imply bias in the OR estimation. Second, the interpretation of our findings might be limited by the sample size. To avoid overfitting due to the sample size, we adopted the best subset selection procedure, which tries to balance the prediction accuracy and the model size. In addition, by including all patients in the largest designated hospital for COVID-19 in Hefei, we believe our study population is representative of patients treated in Hefei, Anhui Province.
